# An eight country cross-sectional study of the psychosocial effects of COVID-19 induced quarantine and/or isolation during the pandemic

**DOI:** 10.1038/s41598-022-16254-8

**Published:** 2022-08-01

**Authors:** Philip J. Schluter, Mélissa Généreux, Elsa Landaverde, Emily Y. Y. Chan, Kevin K. C. Hung, Ronald Law, Catherine P. Y. Mok, Virginia Murray, Tracey O’Sullivan, Zeeshan Qadar, Mathieu Roy

**Affiliations:** 1 School of Health Sciences, University of Canterbury-Te Whare Wananga O Waitaha, Private Bag 4800, Christchurch, 8140 New Zealand; 2 School of Clinical Medicine – Primary Care Clinical Unit, The University of Queensland, Brisbane, 4072 Australia; 3 Department of Community Health Sciences, Faculté de Médecine et des Sciences de la Santé, Université de Sherbrooke, Sherbrooke, Canada; 4 Collaborating Centre for Oxford University and CUHK for Disaster and Medical Humanitarian Response, JC School of Public Health and Primary Care, Chinese University of Hong Kong, Hong Kong, China; 5 Accident and Emergency Medicine Academic Unit, Faculty of Medicine, The Chinese University of Hong Kong, Hong Kong, China; 6 Department of Health, 2932 Manila, Manila Philippines; 7 Collaborating Centre for Oxford University and CUHK for Disaster and Medical Humanitarian Response, JC School of Public Health and Primary Care, Faculty of Medicine, The Chinese University of Hong Kong, Hong Kong, China; 8 UK Health Security Agency, London, UK; 9 Interdisciplinary School of Health Sciences, Faculty of Health Sciences, University of Ottawa, Ottawa, Canada; 10 National Collaborating Centre for Infectious Diseases, Rady Faculty of Health Sciences, University of Manitoba, Winnipeg, Canada; 11 Department of Family Medicine & Emergency Medicine, Faculté de Médecine et des Sciences de la Santé, Université de Sherbrooke, Sherbrooke, Canada

**Keywords:** Risk factors, Signs and symptoms

## Abstract

Forced quarantine and nationwide lockdowns have been a primary response by many jurisdictions in their attempt at COVID-19 elimination or containment, yet the associated mental health burden is not fully understood. Using an eight country cross-sectional design, this study investigates the association between COVID-19 induced quarantine and/or isolation on probable generalized anxiety disorder (GAD) and major depressive episode (MDE) psychological outcomes approximately eight months after the pandemic was declared. Overall, 9027 adults participated, and 2937 (32.5%) were indicated with GAD and/or MDE. Reported quarantine and/or isolation was common, with 1199 (13.8%) confined for travel or health requirements, 566 (6.5%) for being close contact, 720 (8.3%) for having COVID-19 symptoms, and 457 (5.3%) for being COVID-19 positive. Compared to those not quarantining or isolating, the adjusted estimated relative risks of GAD and/or MDE associated with quarantine and/or isolation was significant (*p* < 0.001), ranging from 1.24 (95% confidence interval [CI]: 1.07, 1.43) for travel/health to 1.37 (95% CI 1.19, 1.59) for COVID-19 symptom isolation reasons. While almost universally employed, quarantine and/or isolation is associated with a heavy mental health toll. Preventive strategies are needed, such as minimizing time-limits imposed and providing clear rationale and information, together with additional treatment and rehabilitation resources.

## Introduction

Since declared by the World Health Organization (WHO) as a pandemic^1^, the novel coronavirus disease 2019 (COVID-19) continues to dominate many governmental health, political, economic, and social agendas^2^. Globally, the virus along with its variants rapidly spread and surged in waves, forcing governments to hastily constitute and re-constitute policy balancing competing health and economic imperatives, while operating in a context of fluid and unprecedented uncertainty. As of 3 June 2022, nearly 530 million confirmed cases and 6.3 million deaths across the world had been reported^3^; although with variable reporting practices this is likely an undercount of total deaths attributable to COVID-19^4^. Beyond this human tragedy, the pandemic has decimated economies, ravaged livelihoods, and triggered what is now widely recognized as the most serious global economic crisis since World War II^2^. Stringent conditions to travel, employment, and social engagement have been enforced, while governments pursue varying elimination, containment, and mass vaccination measures. As COVID-19 continues to spread, the impacts of the pandemic further affects populations with these targeted countermeasures increasing and amplifying pre-existing mental and physical health disparities as well as their underlying social determinants within and between nations^5–7^.

Prior to the COVID-19 pandemic, mental health disorders were already among the leading causes of global disability-adjusted life-years, with their importance further increasing in recent years. For instance, depressive and anxiety disorders, which ranked 19 and 34 respectively in 1990, increased to ranks 13 and 24 in 2019^8^. However, the profound and prolonged pandemic directly impacted these and other psychopathologies, regardless of gender, group or region^9,10^. Indeed, in Cénat and colleague’s systematic review and meta-analysis, they found that in populations affected by COVID-19 the prevalence of depression was more than three times and anxiety four times higher than in the pre-COVID-19 general populations observed within a WHO common mental health disorder study^9,11^. The sweeping individual and societal disruptions to people’s life-styles and employment, together with the menace of COVID-19 and its associated psychological demands, may partially explain this phenomenon. These psychological demands were exacerbated by the infodemic—a rapid and far-reaching information overload which includes misinformation and disinformation^12,13^—together with forced quarantine, changes in mental health care service delivery, and nationwide lockdown regimens.

Forced quarantine and nationwide lockdowns are a mainstay for many countries in their COVID-19 response arsenal. So much so that Choukér and Stahn asserted that the world is currently experiencing the largest isolation experiment in history^14^. It is estimated that more than half of the world’s population has experienced some degree of isolation or confinement, through the closure of schools and universities, workplaces, social and physical distancing, and the declaration of health emergencies^9^. While there is variation between countries, managed quarantine facilities have commonly been used for international travelers and COVID-19 positive cases or their close contacts. People with COVID-19 symptoms or with a COVID-19 positive case contact are generally advised to test and self-isolate until result confirmation; self-isolation may also be recommended for others.

Prior to the COVID-19 pandemic, loneliness was so prevalent across Europe, the United States of America (USA), and China that it was described by Jeste and colleagues as a “behavioral epidemic”^15^. Loneliness poses a significant population health problem with increased risk of depression, anxiety, suicidal ideation, negative health behavior, and health care utilization^16^. However, with isolation and confinement restrictions imposed to contain viral spread, the population risk of loneliness and its psychological sequelae steepened. This risk is unequally shared, with older adults, ethnic minorities, those with low income, and those in congregated living environments having a higher risk of loneliness^17,18^. These socially vulnerable groups also have increased pandemic risk^19^. This lead Holt-Lunstad to characterize this pandemic as “the double pandemic of social isolation and COVID-19”^18^. The self-isolation and confinement of large bodies of people for indefinite periods, differences in stay-at-home orders issued by various jurisdictions, and conflicting messages from government and public health authorities have intensified distress^13^. Coupled with increased risk of loneliness, unemployment stresses and financial insecurities, death or infection of family or friends, physical and emotional fatigue, the COVID-19 infodemic, and the impact of COVID-19 contact or diagnosis are all likely to negatively influence the psychological wellbeing of people^9,20^.

Given the complexity of the psychological, social, and neuroscientific effects of COVID-19, mental health and psychosocial impacts are research priorities^21,22^. Convened by the United Kingdom (UK) Academy of Medical Sciences and the mental health research charity, MQ: Transforming Mental Health, one expert panel issued immediate priorities for action and longer-term strategies. Among the immediate priorities are: (i) the collection high-quality data on the mental health effects of the COVID-19 pandemic across the whole population; and, (ii) understanding consequences of the COVID-19 lockdown and social isolation^21^. This panel also saw that multi-discipline international collaboration and a global perspective were beneficial^21^. Convened by the British Psychological Society, another expert panel called for researchers to investigate the immediate and longer-term consequences of COVID-19 for mental health outcomes in the population generally, but also in vulnerable, shielding, and self-isolating groups^22^.

Using an eight country repeated cross-sectional study design, which recruited representative samples of adults, and employed psychometrically robust measures of psychological outcomes, we heed these calls. The overarching goal of this interdisciplinary and international research project was to better understand how risk information was delivered and communicated by authorities and media, and how it was received, understood, and used by the public. Our previous investigation estimated and compared country-specific prevalence of probable generalized anxiety disorder (GAD) and major depressive episode (MDE) at approximately 7 months and 12 months after the earliest COVID-19 case (detected on 17 November 2019, according to unverified media reports on unpublished Chinese government data^23^)^24^. Probable GAD or MDE was indicated by 30.1% and 32.5% of the respondents during the June 2020 and November 2020 measurement waves, respectively, with important variations between countries, gender, and age groups. This study aims to extend these previous findings by investigating the influence of COVID-19 induced quarantine and/or isolation on GAD or MDE psychological outcomes at the November 2020 measurement wave in crude and adjusted analyses, accounting for sociodemographic and potentially confounding variables.

## Methods

### Study design and setting

A cross-sectional study was simultaneously conducted in seven countries (i.e., Canada, USA, England, Switzerland, Belgium, Philippines and New Zealand) and one territory (i.e., Hong Kong) between 6 and 18th November 2020. Although a territory, Hong Kong maintains separate governing and economic systems from that of mainland China and thus, for ease of exposition, it is referred to as a country herein.

### Participants

Adults aged ≥ 18 years residing in one of the eight selected countries at the time of surveying.

### Primary measures

Two psychological measures were utilized, namely: GAD and MDE. These were elicited from the GAD-7 and the Patient Health Questionnaire-9 (PHQ-9) scales, respectively, which are based on the diagnostic criteria described in the Diagnostic and Statistical Manual of Mental Disorders, fourth edition (DSM-IV)^25,26^. The GAD-7 has a composite score ranging from 0–21, while the PHQ-9 score ranges from 0 to 27. For both scales, combined sensitivity and specificity were shown to be maximized at a threshold score of ≥ 10, which is used to identify moderate to severe symptoms of GAD or MDE^25,26^. Thus dichotomous variables were derived, indicated when scores were ≥ 10, and are used to define probable GAD and MDE psychological outcomes, respectively.

Quarantine and/or isolation reasons were derived from several questions. Initially participants were asked: Due to the coronavirus (COVID-19), have you experienced the following disruptions? (i) home quarantine or self-isolation; and (ii) non-home quarantine (e.g. quarantine centre/camp). Each had response options: Yes, No, I don't know/I prefer not to answer. For those responding affirmatively, participants were then asked: For what reason(s) did you have to apply quarantine and/or self-isolation measures? Questions included: (a) COVID-19 diagnosis; (b) COVID-19 symptoms (without diagnosis); (c) exposure to a case of COVID-19; (d) health reason (advanced age, chronic disease, immunosuppression); and, (e) returning from an international trip. Each of these questions also had response options: Yes, No, I don't know/I prefer not to answer. Here, participants were considered to have quarantined and/or isolated if they responded Yes to questions (i) and/or (ii) and Yes to any of (a)-(e). Those who responded Yes to (d) and/or (e) were collapsed into one combined category. For those responding Yes to multiple questions, the question with the highest COVID diagnosis or exposure was used—given by (a)-(e) in descending order.

### Sociodemographic and potential stressor variables

A detailed account of these variables and their definitions appears elsewhere^27^. In brief, gender identity was elicited with response options: male, female, another gender identity, I don't know/I prefer not to answer. Participants responding with “another gender identity” or “I don't know/I prefer not to answer” had their gender set to missing. Age in years was asked, with responses collapsed into 18–24, 25–34, 35–44, 45–54, 55–64, and ≥ 65 years groupings. Usual household composition was elicited and categorized as: living alone, living with others including children, living with others but without children. Participants were questioned as whether they were an essential worker (e.g., healthcare and social services, law enforcement, emergency services, provider of essential goods, educational institution) with response options: yes, no, I don't know/I prefer not to answer. Those who responded affirmatively were asked in which essential sector that that they usually worked, with options: healthcare, social services, law enforcement, emergency services, provider of essential goods, and other sectors. Participants who worked in healthcare and social services were further partitioned from the other essential workers.

The potential stressor variables and threats caused by COVID-19 that are directly related to self were investigated, together with sources and trust in information^24^. Table S1 in the supplementary materials provides the names, descriptions, and response options of all utilized potential stressor variables included in the survey and used here. The survey instrument was validated by the project collaborators, then translated and made available in English, French, German, Italian, and Chinese languages^27^.

### Procedure

A detailed description of the procedure also appears elsewhere^13,27^. Selection of countries for inclusion was based on ensuring global continent diversity within a constrained budget; and capturing different demographics, health systems and policies, and COVID-19 burdens and responses. It was essential for us to have country-specific lead investigators to provided context and ensured the survey was culturally fit-for-purpose. The core team came together from multiple existing professional connections, including the WHO Thematic Platform for Health Emergency and Disaster Risk Management Research Network. As time was of essence and the funding budget limited, the core team purposefully approached potential research leads in identified countries of interest using their pre-existing professional networks, and invited them to opt in. Those who did were included here.

As described previously^13,27^, two polling firms, in collaboration with international partners, undertook recruitment and data collection using an online platform. Participants were randomly recruited from online panels using multiple sources, including traditional and mobile telephone methodologies, social media, and offline methods. To ensure recruitment and representation of hard-to-reach sub-populations, quota sampling was employed. After contact and eligibility confirmation, the purpose, methods of data management, and assurance of confidentiality was fully explained before seeking participant consent in this online study. The survey was designed to take approximately 20 min to complete.

The quota sampling was tailored for each country, and was based on the latest available census population demographics. Strata comprised of age groups (18–24 years, 25–34 years, 35–44 years, 45–54 years, 55–64 years, ≥ 65 years), gender (female, male), and region (which was country-specific). For example, in Canada, regions were defined by Ontario, Québec, British Columbia, Alberta, Manitoba/Saskatchewan, and Atlantic provinces. Table S2 in the supplementary materials provides the stratification variables and values for all eight counties. A 70% minimum recruitment of the estimated stratum numbers for each characteristic (age, gender, and region) was targeted in order to ensure the best possible representation in the sample. This minimum recruitment threshold was a pragmatically determined to maximize participant coverage and scientific robustness while also maximizing cost effectiveness. Survey sampling weights for each country were then calculated in a standardized way^28^. The collected data were then assigned survey sampling weights, correcting for unequal representation, determined from each country’s census and the quotas not being fully achieved, and calibrated to match the sample to population percentage figures for the quota control variables of age groups, gender, and region interlocked.

A minimum sample size target was set at 1000 adults for each participating country, except for Canada (which hosted this research program) which was set at 2000. As outlined earlier^13,27^, three primary core principles and pragmatic considerations were invoked in selecting these sample sizes. They include: (i) largely balanced sample sizes for each country, so investigations of differences between countries have maximal statistical power; (ii) the power of detecting differences in proportions of ≥ 10% or a relative risk of ≥ 1.2 exceeds 80% at the two-tailed α = 0.05 within each country (these detectable differences are moderate to large and likely to be of clinical or meaningful significance); and, (iii) to maximize the number of different countries who were able to participate within a constrained budget.

### Statistical analysis

Reporting of study findings was informed by the STrengthening the Reporting of OBservational studies in Epidemiology (STROBE) guidelines^29^. All analyses were conducted using Stata SE version 17.0 (StataCorp, College Station, TX, USA), accommodated survey sampling weights, employed robust variance estimators, and two-tailed α = 0.05 defined significance.

Participant numbers and sociodemographics by countries were initially described and compared using Pearson’s design-based F-test. Next, to estimate country-specific rates of GAD, MDE, GAD and/or MDE indication, together with their associated 95% confidence intervals (CIs), a binomial regression model with identity link function was employed, treating countries as fixed effects^30,31^.

Recognizing that conventionally employed logistic regression models produce odds ratios with inflated estimates of relative risks (RRs) when the outcome of interest is not rare^32^, an alternative approach was taken. Instead, a modified Poisson regression (with log-link function and robust variance estimators) analysis was used to estimate RRs directly^31,33^. Complete case multilevel mixed-effects Poisson regression models were employed, treating countries as random intercept effects and participants nested within countries, to investigate the association between probable GAD and/or MDE indication and the isolation reason variables. For the crude analysis, only these primary variables were investigated. An adjusted complete-case analysis followed, which considered sociodemographic and potential stressor variables. In the spirit of Sun and colleagues^34^, no variable selection was undertaken for these adjusted analyses.

This adjusted complete-case model was evaluated using the Hosmer–Lemeshow goodness-of-fit test^35^, with the number of groups (*g*) defined by *g* = max(10, min[*m*/2, (*n*–*m*)/2, 2 + 8(*n*/1000)^2^]), where *m* is the number of GAD and/or MDE indications and *n* is the sample size^36^. The area under the receiver operating characteristic (ROC) curve was also used to assess the multivariable model’s predictive ability. A ROC area of 0.5 represents a model with predictive ability that is no better than chance, 0.7–0.8 is considered acceptable, 0.8–0.9 is considered excellent, and more than 0.9 is considered outstanding^35^.

Finally, sensitivity analyses were conducted using multiple imputation (MI) derived from chained equations using all variables within the multivariable models. M = 50 replications were generated and analyzed, with coefficients and standard errors for the variability between imputations combined according to Rubin’s rule^37^. Differences in estimated effect sizes and ROC areas between imputed and complete case analyses were then derived and reported.

### Ethics

This study sits within a broader program of research funded by the Canadian Institutes of Health Research, reviewed and approved by the Research Ethics Board of the CIUSSS de l’Estrie—CHUS (HEC ref: 2020–3674). Informed consent was obtained from all participants before their participation, and the collection of information was carried out confidentially. Participants were able to withdraw at any time without penalty or need for explanation. The datasets did not carry any personally identifiable information. The study complied with the ethical standards for human experimentation as established by the Helsinki Declaration and Canada’s HEC. All methods and reporting were performed in accordance with HEC’s relevant guidelines and regulations.

## Results

### Participants and their characteristics

Overall, 9027 adults participated in the survey (Canada: 2004; USA: 1003; England: 1000: Belgium: 1014; Switzerland: 1000; Hong Kong: 1002; Philippines: 1003; and, New Zealand: 1001). Their mean age was 47.0 years (standard deviation 17.0 years; range 18–99 years), 52.0% were female (42 [0.5%] did not identify as being female or male), 26.7% classified themselves as being essential workers (of whom, 34.1% were health workers), and 28.7% lived in households with children. The weighted numbers for participants’ demographic characteristics overall and by country appear in Table 1.

**Table 1:** Sample numbers and weighted distribution of respondents’ demographic characteristics, overall and partitioned by country.

Country	N	Age (yr)	Female^a^	Essential worker^b^			Household composition		
				No	Yes: health	Yes: other	Alone	With child(ren)^n^	Other
		mean (SD)	n (%)	n (%)	n (%)	n (%)	n (%)	n (%)	n (%)
Canada	2004	47.8 (17.2)	1,031 (51.7)	1,452 (73.9)	176 (8.9)	338 (17.2)	366 (18.3)	449 (22.4)	1,189 (59.3)
USA	1003	48.4 (17.2)	517 (51.9)	720 (73.1)	90 (9.2)	174 (17.7)	230 (22.9)	303 (30.2)	470 (46.9)
England	1000	47.6 (17.1)	511 (51.2)	730 (74.8)	78 (8.0)	168 (17.2)	212 (21.2)	257 (25.7)	532 (53.2)
Belgium	1014	49.4 (16.2)	520 (51.6)	766 (77.6)	60 (6.1)	161 (16.3)	190 (18.8)	227 (22.4)	597 (58.9)
Switzerland	1000	50.1 (17.1)	522 (52.2)	772 (78.9)	97 (9.9)	111 (11.3)	271 (27.1)	185 (18.5)	544 (54.4)
Hong Kong	1002	46.5 (15.7)	550 (55.0)	606 (62.3)	107 (11.0)	260 (26.7)	67 (6.7)	279 (27.8)	656 (65.5)
Philippines	1003	38.1 (14.7)	503 (50.7)	676 (71.0)	104 (11.0)	171 (18.0)	34 (3.4)	552 (55.0)	417 (41.6)
New Zealand	1001	46.9 (17.7)	513 (51.4)	729 (74.5)	87 (8.9)	162 (16.5)	156 (15.6)	341 (34.0)	505 (50.4)
Total	9027	47.0 (17.0)	4667 (52.0)	6450 (73.3)	800 (9.1)	1544 (17.6)	1526 (16.9)	2591 (28.7)	4910 (54.4)

^a^42 (0.5%) respondents did not identify as being male or female; ^b^234 (2.6%) did not know or declined to answer.

The survey sample weightings ensured that reported numbers were representative of the current sociodemographic profile for each country. Notable differences in these demographic characteristics appeared between countries. For instance, Filipino participants were on average younger and more likely to live in households with children than participants from other countries, whereas Swiss participants were older, less likely to be an essential worker, and more likely to be living alone; see Table 1.

### Psychological outcomes

**Figure 1:**
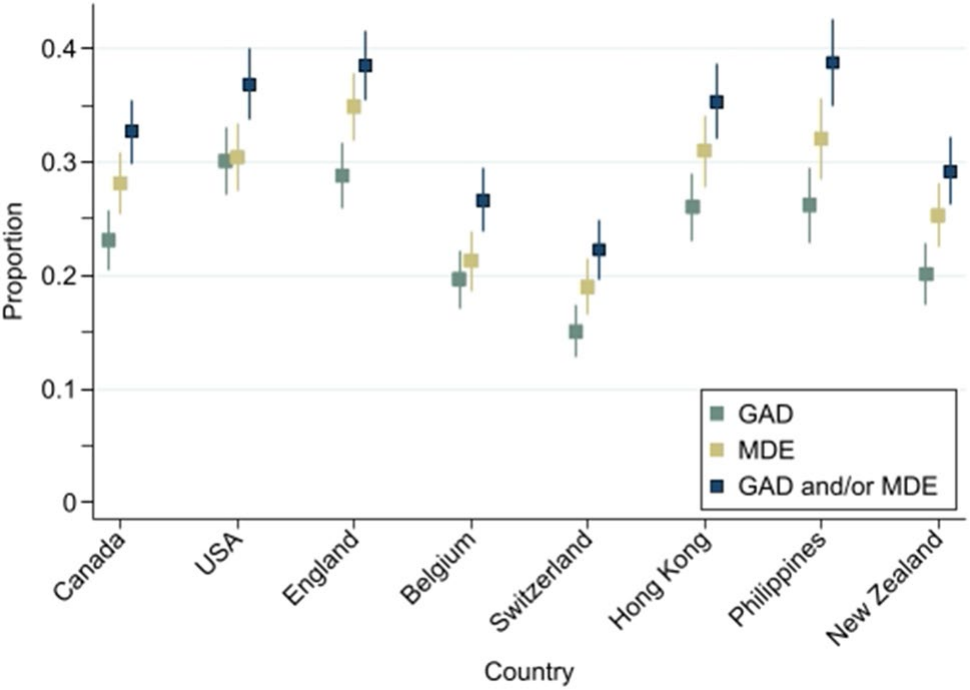
Proportion of participants indicated for probable generalized anxiety disorder (GAD), major depression episode (MDE), and GAD and/or MDE, together with associated 95% confidence intervals (CIs) for the eight participating countries/regions.

Approximately 12 months after the first COVID-19 case was detected in Wuhan, China, probable GAD was indicated by 2129 (23.6%) participants, probable MDE was indicated by 2509 (27.8%), while 2937 (32.5%) were indicated with GAD and/or MDE. Of the 2937 participants indicated, 57.9% were indicated for both, 14.6% were indicated for probable GAD but not MDE, and 27.5% were indicated for probable MDE but not GAD, a significant asymmetry (*p* < 0.001). From a fixed effects binomial regression model, significant differences emerged in the rates of these psychological outcomes between countries (all *p* < 0.001). Figure 1 presents these rates, together with 95% CIs, for the eight countries.

Estimated rates of probable GAD indication ranged from 15.6% (95% CI 12.7%, 17.4%) in Switzerland to 30.1% (95% CI 27.1%, 33.1%) in the USA; probable MDE indication ranged from 19.0% (95% CI 16.5%, 21.5%) in Switzerland to 34.9% (95% CI 31.9%, 37.9%) in England; and, for GAD and/or MDE indication, rates ranged from 22.3% (95% CI 19.6%, 24.9%) in Switzerland to 38.8% (95% CI 35.0%, 42.5%) in the Philippines; see Fig. 1.

### Quarantine and/or isolation reason

In total, 8695 (96.3%) participants responded to questions relating to COVID-19 related quarantine and/or isolation. At the time of the survey, 5753 (66.2%) respondents had not quarantined or isolated, 1199 (13.8%) had quarantined and/or isolated for travel or health requirements, 566 (6.5%) quarantined and/or isolated because they were a close contact for a COVID case, 720 (8.3%) isolated because of COVID-19 symptoms, and 457 (5.3%) respondents quarantined and/or isolated in response to a positive COVID-19 case diagnosis. Again, significant differences in these distributions emerged between countries (*p* < 0.001). Any reported quarantined and/or isolation reason was highest among USA (41.3%) and Filipino (41.3%) participants, and lowest among those from Hong Kong (23.1%) and New Zealand (28.0%); see Table 2.

**Table 2:** Weighted distribution of participant’s response to COVID-19 induced quarantine and/or isolation questions overall and partitioned by country.

Country	No isolation	Travel/health	COVID contact	COVID symptoms	COVID diagnosis
	n (%)	n (%)	n (%)	n (%)	n (%)
Canada	1266 (65.5)	338 (17.5)	96 (5.0)	206 (10.7)	26 (1.3)
USA	563 (58.7)	168 (17.5)	80 (8.3)	69 (7.2)	78 (8.2)
England	624 (65.8)	137 (14.5)	58 (6.1)	68 (7.2)	61 (6.4)
Belgium	675 (68.2)	95 (9.6)	70 (7.1)	93 (9.4)	56 (5.7)
Switzerland	631 (64.5)	140 (14.3)	98 (10.0)	63 (6.4)	47 (4.8)
Hong Kong	732 (76.9)	59 (6.2)	46 (4.8)	37 (3.9)	79 (8.3)
Philippines	557 (58.3)	125 (13.1)	94 (9.9)	89 (9.3)	92 (9.6)
New Zealand	705 (72.0)	137 (13.9)	24 (2.4)	95 (9.7)	20 (2.0)
Total	5753 (66.2)	1199 (13.8)	566 (6.5)	720 (8.3)	457 (5.3)

332 (3.7%) participants had missing data for quarantine and/or isolation questions.

### Crude analyses

**Figure 2:**
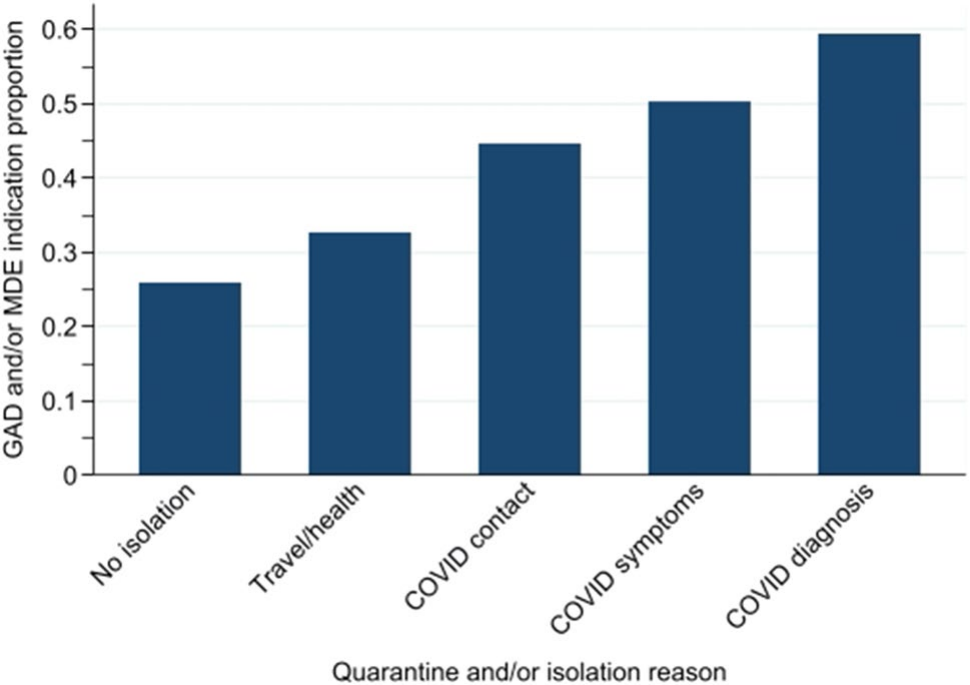
Proportion of participants indicated for probable generalized anxiety disorder (GAD) and/or major depression episode (MDE) over quarantine and/or isolation reason categories.

The proportion of participants indicated for probable GAD and/or MDE over quarantined and/or isolation reason categories appears in Fig. 2. Evident from Fig. 2 is the increased proportion indicated with increased COVID-19 exposure and diagnosis; ranging from 26.0% for participants who have not quarantined or isolated to 59.4% for those who quarantined and/or isolated due to having a COVID-19 diagnosis.

In a multilevel mixed-effects Poisson model, treating countries as random effects and participants nested within countries, this observed pattern was significant (*p* < 0.001). Table 3 gives the distribution of probable GAD and/or MDE indication by quarantine and/or isolation reason categories, together with RRs and associated 95% CIs estimates from this crude analysis. Compared to participants who did not quarantine or isolate, those who quarantined and/or isolated in response to a positive COVID-19 diagnosis had increased risk of probable GAD and/or MDE indication estimated at 2.22 (95% CI 1.80, 2.75); see Table 3. Among participants who quarantined and/or isolated: those with COVID contact had significantly higher risk of probable GAD and/or MDE indication compared to those confined for travel/health reasons (*p* < 0.001); those with COVID symptoms had significantly higher risk of probable GAD and/or MDE indication than those with COVID contact (*p* = 0.04); but, those with COVID diagnosis had risk of probable GAD and/or MDE indication not significantly higher than those with COVID symptoms (*p* = 0.18). In this model, the variance component associated with the country random effect was estimated at σ = 0.173 (95% CI 0.097, 0.307).

**Table 3:** Distribution of probable GAD and/or MDE indication by quarantine and/or isolation reason categories, together with relative risks (RRs) and associated 95% confidence intervals (CIs) estimates from crude and adjusted complete case multilevel logistic models, and the multiple imputed (MI) adjusted multilevel logistic model.

Quarantine and/or isolation reason	N	GAD/MDE	Crude^a^	Adjusted^b,c^	MI adjusted^c^
		n (%)	RR (95% CI)	RR (95% CI)	RR (95% CI)
No isolation	5753	1493 (26.0)	1.00 (reference)	1.00 (reference)	1.00 (reference)
Travel/health	1199	392 (32.7)	1.25 (1.09, 1.43)	1.24 (1.07, 1.43)	1.22 (1.07, 1.40)
COVID contact	566	253 (44.7)	1.74 (1.53, 1.96)	1.27 (1.12, 1.45)	1.25 (1.11, 1.41)
COVID symptoms	720	362 (50.2)	1.94 (1.70, 2.20)	1.37 (1.19, 1.59)	1.38 (1.21, 1.57)
COVID diagnosis	457	272 (59.4)	2.22 (1.80, 2.75)	1.32 (1.20, 1.46)	1.33 (1.18, 1.49)

^a^332 (3.7%) respondents missing; ^b^1097 (12.2%) respondents missing; ^c^adjusted for sex, age, essential worker, household composition, financial losses, threat perceived to oneself and/or family, threat perceived for country and/or world, being a victim of stigma, level of information about COVID-19, trust in authorities score, social networks used as a regular source of information, friend/family/co-workers as a regular source of information, sense of coherence.

### Adjusted complete case analyses

Results from the multivariable multilevel mixed-effects Poisson model, adjusted for sex, age, essential worker, household composition, financial losses, threat perceived to oneself and/or family, threat perceived for their country and/or world, being a victim of stigma, level of information about COVID-19, trust in authorities score, social networks used as a regular source of information, friend/family/co-workers as a regular source of information, and sense of coherence, also appears in Table 3 and Table S3 within the supplementary materials. Table S3 gives the distribution of probable GAD and/or MDE indication for considered sociodemographic and potential stressor variables together with RRs and associated 95% CIs estimates from crude and adjusted complete case multilevel Poisson models. Complete data for all considered variables were available from 7930 (87.8%) participants.

Although dampened, the relationship between GAD and/or MDE indication and quarantine and/or isolation reason remained significant (*p* < 0.001). Compared to participants who did not quarantine or isolate, those who quarantined and/or isolated in response to a positive COVID-19 diagnosis had increased adjusted risk of probable GAD and/or MDE indication estimated at 1.32 (95% CI 1.20, 1.46); see Tables 3 and S3. However, amongst the people who had quarantined and/or isolated, there was no significant difference in any pairwise comparison groups (all *p* > 0.05).

All considered sociodemographic and potential stressor variables were significant within the multivariable model except for: threat perceived for their country and/or world (*p* = 0.49); friend/family/co-workers as a regular source of information (*p* = 0.09); and, essential worker (*p* = 0.09). Also noteworthy is that young adults, those with a weaker sense of coherence, and those unsure or unknown about their financial losses had relatively high estimated adjusted RRs; see Table S3. Unlike the crude analysis, there was no difference in the estimated RRs between groups for those participants who quarantined and/or isolated in any pairwise comparison (all *p* > 0.05). The variance component associated with the country random effect in this model was estimated at σ = 0.147 (95% CI 0.090, 0.240). In terms of regression diagnostics, this complete case multivariable model yielded a Hosmer–Lemeshow goodness-of-fit *p* = 0.99 (based on *g* = 505) and the AUC = 0.789 (95% CI 0.779, 0.800), a value which is considered acceptable. This evidence suggests that the model had adequate fit.

### Sensitivity analyses

After undertaking chained equations MI for missing data (M = 50), and repeating the multivariable multilevel mixed-effects logistic regressions, the resulting estimates were strikingly similar to those derived from the complete case analyses; see Tables 3 and S3. In terms of absolute change in the estimated adjusted RR between the primary variables of interest, the greatest shift occurred for those in the COVID contact category—moving from 1.27 (95% CI 1.12, 1.45) to 1.25 (95% CI 1.11, 1.41); a negligible difference. The mean estimated ROC area for these M = 50 multiple imputations was 0.786 (95% CI 0.776, 0.796); again, this is similar to the complete case estimate and a level that continues to represent acceptable predictive accuracy.

## Discussion

COVID-19 induced quarantine and/or isolation is common^9,14^. Over one in three participants in this study reported they had quarantined and/or isolated due to having a COVID-19 diagnosis, symptoms, being a close contact, or through travel/health reasons at some time during the approximate 12 months since the first COVID-19 case was detected and eight months after the WHO declared a pandemic^1,23^. There were significant differences between countries—with quarantine and/or isolation rates higher for the USA (which had a relatively high cumulative death rate of 73.9 per 100,000 people) and lower for Hong Kong (cumulate death rate of 1.45 per 100,000 people) and New Zealand (cumulate death rate of 0.52 per 100,000 people)^3^. Yet, as the virus continues to spread and surge, these quarantine and/or isolation rates will only increase—as, for example, has been witnessed recently in New Zealand^38^.

It is known that periods of isolation, even for relatively short durations (< 10 days), can have significant and enduring negative psychological and psychiatric effects^39,40^. Common psychological disorders include depressive symptoms and post-traumatic stress, anxiety and panic, obsessive–compulsive symptoms, insomnia, and digestive problems among others^41^. This effect is likely compounded by the increased population mental health burden which follows infectious disease outbreaks and natural disasters^42,43^. Therefore, the current global COVID-19 pandemic and the extent of quarantined and/or isolation as primary response by many countries is likely to have significant, potentially unprecedented, burden. However, currently, there is relatively little empirically known on the measured extent of quarantine and/or isolation on mental health in this current pandemic, hence the calls from the expert panels^21,22^.

GAD and/or MDE was indicated by 32.5% of participants in this sample; a prevalence that has increased over time^13^. Considerable variability in GAD and/or MDE indication rates between countries was observed. This, in part, likely reflects the different contextually-specific countermeasures applied by governments and authorities^13,39^. Elements, such as their level of severity, length of implementation, and how they are communicated, are known to be important. For instance, confusion from poorly coordinated public health messages and strategies between jurisdictions and levels of government has previously been associated with observed negative psychological outcomes in the USA and Canada^39^; and seen here^13^. Moreover, the lockdown length enforced in the Philippines (spanning nearly six months at the time of the measurement wave) likely contributed to the highest levels of observed GAD and/or MDE^44^. Conversely, Belgium and Switzerland lockdowns were less rigid than other counterpart countries. With their businesses and restaurants remaining open (albeit with restrictions), this likely engendered a sense of relative normalcy and mitigated against the psychological impacts of the pandemic^13^.

Crude analysis showed a marked ‘dose–response’ whereby those with increased COVID-19 case likelihood also had increased GAD and/or MDE indication. However, in adjusted complete case and MI analyses, no gradient was evinced. After controlling for a range of sociodemographic and potential stressor variables, those who quarantined and/or isolated had risk of GAD and/or MDE indication that was 20–40% higher than those who did not have COVID-19 related quarantine or isolation. Although non-significant, the estimated effect size was marginally higher for those quarantining and/or isolating with COVID-19 symptoms compared to those with cases is of interest, and suggests that the uncertainty of their case status may contribute^45^. It is notable in the adjusted analyses that people who were younger and had a weaker sense of coherence, in particular, were at increased GAD and/or MDE, over and above the quarantine and/or isolation effect.

In a rapid review, Brooks and colleagues identified stressors of quarantine which included duration, infection fears, frustration, boredom, inadequate supplies, inadequate information, financial loss, and stigma^39^. The infodemic, that overloads individuals with information, also acts as a stressor—particularly among the younger, social media savvy users and consumers as the information is rapidly shared and is often misinformation and disinformation^12,24,45^. False information, perceived as novel and fresh, spreads more quickly and widely than true information^46^. This fuels confusion, uncertainty and anger, contributing to greater mental health risk^12,13,47^. There is also a well-recognized social gradient of risk, which emerges from the interaction between social determinants of health, risk of exposure, and adverse impacts from a pandemic^48^. Quarantine and isolation, coupled with their accompanying stressors, thus likely triggers mental illness for some; a response that is unequally shared across populations. Strategies designed to mitigate the effect of quarantine and isolation need to be cognizant of these stressors, and people’s varied vulnerability profiles and responses^48^.

Our findings do not diminish the role of quarantine and isolation, as these measures have been a centuries-old cornerstone for the successful public health response to emerging and re-emerging infectious diseases^49^. Indeed, in this COVID-19 era, combinations of non-pharmaceutical interventions which include quarantine and isolation have been shown to have the greatest effect on virus containment^50^. However, the effectiveness of these intervention combinations depend on their local context, such as timing of their adoption, and carry different levels of adverse individual and societal impacts^50^.

### Strengths and limitations

While having notable strengths, such as the relatively large and balanced sample size across countries (except Canada), timeliness of recruitment, spread of participants across eight countries and four continents, use of a psychometrically robust instruments for psychological distress indication, and the careful data analysis, this study also has limitations. As asserted previously^13,27^, arguably the sampling mechanism and associated unmeasurable non-sampling bias potentially represents the biggest threat to the study’s validity. There were a number of pragmatic considerations in designing, attracting funding, securing ethics, and implementing this international study within a relatively short time frame^13,27^. In particular, there were competing tensions in maximizing expedited cost-effective data collection processes and international reach while simultaneously minimizing non-sampling bias. Our adopted study design sought to balance these competing demands, without compromising scientific rigour or the production of high quality data. Participants were randomly recruited from data sources derived from various online and offline panels; a modern but less conventional research sampling frame method^28^. The implemented quota sampling, together with survey sampling weight adjustments, was designed to ensure that the sample was approximately representative of the target adult population within each country. However, some population groups are likely to be underrepresented, such as people having limited or no internet access, those with lower or poor literacy attainment, or those living with disabilities or mental illnesses^51^. If these underrepresented groups have a differential pattern of response compared those included within the study, then bias may be introduced despite the sampling and weighting methods employed. Another limitation is the cross-sectional design. Quarantine and/or isolation reasons were only elicited in the November 2020 measurement wave, and not in the June 2020 measurement wave^13^. Elicitation at both waves would have introduced a useful temporal element and assisted in providing evidence as to whether the effects observed here were consistent or differentially affected over time. Moreover, it should also be noted that the mental health impacts of the pandemic, and the nature of quarantine and isolation measures, are dynamic and rapidly evolving—so that relationships and effect sizes reported here may not accurately reflect those observed in the future. Also, this study investigates the mental health effects associated with just one suite of non-pharmaceutical interventions, namely: quarantine and isolation. However, interventions were often used in combinations, and these other interventions may have contributed to additional mental health burdens which have not been separated out^50^. Although, the eight country design of this study, each having different combinations and timing of interventions, is likely to mitigate this limitation. Finally, the adjusted modelling may have suffered from residual or unmeasured confounding effects. Unmeasured confounding variables can result in substantial bias in the estimated exposure-outcome adjusted RR, particularly if it is uncorrelated with the measured explanatory variables^52^. Study replication using different suites of variables is needed to understand its effect.

## Conclusions

Globally, the COVID-19 pandemic era represents an extraordinary time for most societies; a time which necessitated extraordinary responses that come at individual and societal costs. While commonly employed, forced quarantine and nationwide lockdowns carries such a cost. To minimize the impact of quarantine and isolation, officials should quarantine individuals for no longer than required, provide clear rationale for quarantine accompanied by information about protocols, provide sufficient supplies, and strive to ensure the experience is as tolerable as possible^39,40^. It is imperative to recognize the extent and magnitude of mental health issues; quarantine and isolation preventive strategies should be adopted where possible. Nonetheless, given the large and increasing number of people involved, governments are obligated to instigate clear mental health service provision strategies to cater for the inevitable additional treatment and rehabilitation needs following their primary COVID-19 quarantine and isolation response.

## Supplementary Information

Supplementary Information.

## Data Availability

The datasets used for statistical analysis are held by M.G. at the Faculté de Médecine et des Sciences de la Santé, Université de Sherbrooke, Sherbrooke, Canada. Application to use these data must be made to M.G. through the corresponding author.
