# Supplementary material for: An eight country cross-sectional study of the psychosocial effects of COVID-19 induced quarantine and/or isolation during the pandemic
Source: Sci Rep. 2022 Aug 1;12:13175. doi: 10.1038/s41598-022-16254-8 (PMC9341149; doi:10.1038/s41598-022-16254-8)
Supplement: Supplementary file 1 — Supplementary Information. [file 41598_2022_16254_MOESM1_ESM.docx]

**Supplementary materials for:**

**An eight country cross-sectional study of the psychosocial effects of COVID-19 induced quarantine and/or isolation during the pandemic**

**Table S1.** Names, descriptions and response options for the considered potential stressor variables.

| **Name** | | **Descriptions (response options)** |
| --- | --- | --- |
| *Financial losses* | | |
|  | Having experienced financial losses of any kind due to COVID-19 (yes, no, unsure/ unknown) | |
| *Threat perceived for oneself and/or family* | | |
|  | Level of threat posed by the COVID-19 perceived for oneself and/or the family (very low, low, moderate, high, very high) | |
| *Threat perceived for country and/or world* | | |
|  | Level of threat posed by the COVID-19 perceived for the country and/or the world (very low, low, moderate, high, very high) | |
| *Being a victim of stigma* | | |
|  | Being a victim of stigma or discrimination due to the COVID-19 (yes, no, decline to answer). | |
| *Level of information about COVID-19* | | |
|  | Level would you rank your level of information about the COVID-19 (10-point scale ranging from: 1 is a very low level, to 10 is a very high level; higher defined as (9-10), lower as (1-8)) | |
| *Trust in authorities score* | | |
|  | Level would you rank your level of trust in (1) scientists, doctors and health experts; (2) national health organizations; (3) global health organizations; (4) government (each with 10-point scale ranging from: 1 is a very low level, to 10 is a very high level). These four scores were summed, and partitioned into approximate quartiles based on the observed response distribution. | |
| *Internet-based social media* *as a regular source of information* | | |
|  | Extent that social networks (e.g., Facebook, Twitter, other networks, Instagram, etc.) used to inform yourself about the COVID-19 (mainly/always, often, sometimes, not much/never) | |
| *Friends/family/co-workers as a regular source of information* | | |
|  | Extent that friends/family/co-workers used to inform yourself about the COVID-19 (mainly/always, often, sometimes, not much/never) | |
| *Sense of coherence* | | |
|  | Measured using the three item Sense of Coherence (SOC-3) instrument [1, 2], corresponding to comprehensibility, manageability, and meaningfulness. Here, participants were asked (1) Do you usually see a solution to problems and difficulties that other people find hopeless?; (2) Do you usually feel that your daily life is a source of personal satisfaction?; (3) Do you usually feel that the things that happen to you in your daily life are hard to understand? (each with response options: no (0), yes - sometimes (1), yes - usually (2). Question (3) was reverse scored, then the three scores summed and dichotomized using the threshold: weaker (summed score of 0-4) or stronger (summed score of 5-6)) | |

**Table S2**. Stratification variables and values used for sample selection over all participating eight counties.

|  | **Canada** | **USA** | **England** | **Belgium** | **Switzerland** | **Hong Kong** | **Philippines** | **New Zealand** |
| --- | --- | --- | --- | --- | --- | --- | --- | --- |
| *Gender* | |  |  |  |  |  |  |  |
|  | Female | Female | Female | Female | Female | Female | Female | Female |
|  | Male | Male | Male | Male | Male | Male | Male | Male |
| *Age groups (years)* | | |  |  |  |  |  |  |
|  | 18-24 | 18-24 | 18-24 | 18-24 | 18-24 | 18-24 | 18-24 | 18-24 |
|  | 25-34 | 25-34 | 25-34 | 25-34 | 25-34 | 25-34 | 25-34 | 25-34 |
|  | 35-44 | 35-44 | 35-44 | 35-44 | 35-44 | 35-44 | 35-44 | 35-44 |
|  | 45-54 | 45-54 | 45-54 | 45-54 | 45-54 | 45-54 | 45-54 | 45-54 |
|  | 55-64 | 55-64 | 55-64 | 55-64 | 55-64 | 55-64 | 55-64 | 55-64 |
|  | ≥65 | ≥65 | ≥65 | ≥65 | ≥65 | ≥65 | ≥65 | ≥65 |
| *Region* | |  |  |  |  |  |  |  |
|  | Ontario | New England | East | Région de Bruxelles-Capitale | Zurich | Hong Kong Island | National capital region | Northland |
|  | Québec | Middle Atlantic | East Midlands | Anvers | Bern | Kowloon | Cordillera administrative region | Auckland |
|  | British Columbia | East North Central | London | Flandre orientale | Lucerne | New Territories | Ilocos | Waikato |
|  | Alberta | West North Central | North East | Flandre occidentale | Uri |  | Cagayan valley | Bay of Plenty |
|  | Manitoba/ Saskatchewan | South Atlantic | North West | Limbourg | Schwyz |  | Central luzon | Gisborne |
|  | Atlantic | East South Central | South East | Brabant flamand | Obwalden |  | Calabarzon | Hawke’s Bay |
|  |  | West South Central | South West | Brabant Wallon | Nidwalden |  | Mimaropa region | Taranaki |
|  |  | Mountain | West Midlands | Hainaut | Glarus |  | Bicol | Manawat |
|  |  | Pacific | Yorkshire and the Humber | Liège | Zoug |  | Western visayas | Wellington |
|  |  |  |  | Namur | Fribourg |  | Central visayas | Tasman |
|  |  |  |  | Luxembourg | Solothurn |  | Eastern visayas | Nelson |
|  |  |  |  |  | Basel city |  | Zamboanga peninsula | Marlborough |
|  |  |  |  |  | Basel-Landschaft |  | Northern mindanao | West Coast |
|  |  |  |  |  | Schaffhausen |  | Davao | Canterbury |
|  |  |  |  |  | Appenzell Rh.-Ext. |  | Soccsksargen | Otago |
|  |  |  |  |  | Appenzell Rh.-Int. |  | Autonomous region in muslim mindanao | Southland |
|  |  |  |  |  | Saint Gall |  |  |  |
|  |  |  |  |  | Grisons |  |  |  |
|  |  |  |  |  | Argovia |  |  |  |
|  |  |  |  |  | Thurgau |  |  |  |
|  |  |  |  |  | Ticino |  |  |  |
|  |  |  |  |  | Vaud |  |  |  |
|  |  |  |  |  | Valais |  |  |  |
|  |  |  |  |  | Neuchatel |  |  |  |
|  |  |  |  |  | Geneva |  |  |  |
|  |  |  |  |  | Jura |  |  |  |

*Regions used for weighting of the data were determined from the most recent census data of each country.

**Table S3**. Distribution of probable GAD and/or MDE indication for sociodemographic and potential stressor variables together with relative risks (RRs) and associated 95% confidence intervals (CIs) estimates from crude and adjusted complete case multilevel Poisson models, and the multiple imputed (MI) adjusted multilevel Poisson model.

|  |  |  | **GAD/MDE** | **Crude^a^** | **Adjusted^b^** | **MI adjusted** |
| --- | --- | --- | --- | --- | --- | --- |
|  | | N | n (%) | RR (95% CI) | RR (95% CI) | RR (95% CI) |
| *Isolation reason* | |  |  |  |  |  |
|  | No isolation | 5,753 | 1,493 (26.0) | 1.00 (reference) | 1.00 (reference) | 1.00 (reference) |
|  | Travel/health | 1,199 | 392 (32.7) | 1.25 (1.09, 1.43) | 1.24 (1.07, 1.43) | 1.22 (1.07, 1.40) |
|  | COVID contact | 566 | 253 (44.7) | 1.74 (1.53, 1.96) | 1.27 (1.12, 1.45) | 1.25 (1.11, 1.41) |
|  | COVD symptoms | 720 | 362 (50.2) | 1.94 (1.70, 2.20) | 1.37 (1.19, 1.59) | 1.38 (1.21, 1.57) |
|  | COVID diagnosis | 457 | 272 (59.4) | 2.22 (1.80, 2.75) | 1.32 (1.20, 1.46) | 1.33 (1.18, 1.49) |
| *Sex* | |  |  |  |  |  |
|  | Male | 4,318 | 1,323 (30.6) | 1.00 (reference) | 1.00 (reference) | 1.00 (reference) |
|  | Female | 4,667 | 1,596 (34.2) | 1.12 (1.04, 1.20) | 1.14 (1.06, 1.23) | 1.12 (1.04, 1.20) |
| *Age (years)* | |  |  |  |  |  |
|  | 18-24 | 995 | 550 (55.2) | 3.97 (2.68, 5.89) | 2.54 (1.84, 3.51) | 2.52 (1.84, 3.44) |
|  | 25-34 | 1,657 | 745 (45.0) | 3.24 (2.21, 4.76) | 2.12 (1.54, 2.91) | 2.12 (1.55, 2.90) |
|  | 35-44 | 1,468 | 555 (37.8) | 2.70 (1.89, 3.87) | 1.89 (1.41, 2.53) | 1.89 (1.41, 2.52) |
|  | 45-54 | 1,657 | 540 (32.6) | 2.35 (1.71, 3.23) | 1.73 (1.34, 2.24) | 1.76 (1.36, 2.29) |
|  | 55-64 | 1,467 | 303 (20.7) | 1.49 (1.07, 2.08) | 1.30 (0.98, 1.73) | 1.32 (1.00, 1.75) |
|  | ≥65 | 1,783 | 245 (13.7) | 1.00 (reference) | 1.00 (reference) | 1.00 (reference) |
| *Essential worker* | |  |  |  |  |  |
|  | No | 6,450 | 1,887 (29.3) | 1.00 (reference) | 1.00 (reference) | 1.00 (reference) |
|  | Yes: health | 800 | 334 (41.7) | 1.41 (1.30, 1.53) | 1.03 (0.89, 1.19) | 1.04 (0.92, 1.17) |
|  | Yes: other | 1,544 | 610 (39.5) | 1.32 (1.18, 1.49) | 1.08 (1.01, 1.16) | 1.07 (1.02, 1.13) |
| *Household composition* | | |  |  |  |  |
|  | Alone | 1,526 | 446 (29.2) | 1.01 (0.91, 1.12) | 1.10 (1.06, 1.14) | 1.07 (1.04, 1.11) |
|  | With children | 2,591 | 1,039 (40.1) | 1.33 (1.17, 1.51) | 1.08 (1.01, 1.16) | 1.08 (1.00, 1.17) |
|  | Other | 4,910 | 1,452 (29.6) | 1.00 (reference) | 1.00 (reference) | 1.00 (reference) |
| *Financial losses* | |  |  |  |  |  |
|  | No | 4,783 | 1,142 (23.9) | 1.00 (reference) | 1.00 (reference) | 1.00 (reference) |
|  | Yes | 3,875 | 1,639 (42.3) | 1.77 (1.63, 1.91) | 1.31 (1.18, 1.46) | 1.29 (1.17, 1.43) |
|  | Unsure/unknown | 370 | 157 (42.5) | 1.76 (1.43, 2.17) | 1.51 (1.32, 1.74) | 1.35 (1.09, 1.67) |
| *Threat perceived to oneself and/or family* | | | |  |  |  |
|  | Higher | 3,420 | 1,456 (42.6) | 1.62 (1.45, 1.80) | 1.40 (1.26, 1.54) | 1.39 (1.27, 1.51) |
|  | Lower | 5,334 | 1,391 (26.1) | 1.00 (reference) | 1.00 (reference) | 1.00 (reference) |
| *Threat perceived for country and/or world* | | | |  |  |  |
|  | Higher | 6,553 | 2,203 (33.6) | 1.17 (0.96, 1.43) | 1.05 (0.91, 1.22) | 1.07 (0.94, 1.22) |
|  | Lower | 2,179 | 636 (29.2) | 1.00 (reference) | 1.00 (reference) | 1.00 (reference) |
| *Being a victim of stigma* | | |  |  |  |  |
|  | No | 7,498 | 2,110 (28.1) | 1.00 (reference) | 1.00 (reference) | 1.00 (reference) |
|  | Yes | 1,107 | 660 (59.6) | 2.10 (1.76, 2.50) | 1.31 (1.20, 1.44) | 1.35 (1.25, 1.46) |
|  | Decline to answer | 422 | 167 (39.6) | 1.39 (1.13, 1.71) | 0.90 (0.72, 1.12) | 0.89 (0.74, 1.08) |
| *Level of information about COVID-19* | | | |  |  |  |
|  | Higher (9-10) | 2,793 | 948 (33.9) | 1.00 (reference) | 1.00 (reference) | 1.00 (reference) |
|  | Lower (1-8) | 6,234 | 1,990 (31.9) | 0.96 (0.93, 1.00) | 0.91 (0.84, 0.98) | 0.92 (0.87, 0.97) |
| *Trust in authorities score* | | | |  |  |  |
|  | Q1 (low) | 2,355 | 885 (37.6) | 1.33 (1.17, 1.51) | 1.25 (1.17, 1.34) | 1.26 (1.16, 1.35) |
|  | Q2 | 1,981 | 668 (33.7) | 1.20 (1.08, 1.34) | 1.15 (1.03, 1.29) | 1.14 (1.00, 1.30) |
|  | Q3 | 2,257 | 675 (29.9) | 1.05 (0.95, 1.16) | 1.03 (0.95, 1.12) | 1.06 (0.95, 1.17) |
|  | Q4 (high) | 2,434 | 709 (29.1) | 1.00 (reference) | 1.00 (reference) | 1.00 (reference) |
| *Social networks used as a regular source of information* | | | | |  |  |
|  | Often/always | 2,652 | 1,145 (43.2) | 1.54 (1.29, 1.83) | 1.19 (1.07, 1.32) | 1.16 (1.04, 1.30) |
|  | Sometimes/never | 5,990 | 1,669 (27.9) | 1.00 (reference) | 1.00 (reference) | 1.00 (reference) |
| *Friend/family/co-workers as a regular source of information* | | | | |  |  |
|  | Often/always | 3,752 | 1,321 (35.2) | 1.13 (1.02, 1.26) | 0.93 (0.85, 1.01) | 0.94 (0.88, 1.01) |
|  | Sometimes/never | 5,091 | 1,544 (30.3) | 1.00 (reference) | 1.00 (reference) | 1.00 (reference) |
| *Sense of coherence* | | | |  |  |  |
|  | Stronger (5-6) | 2,719 | 332 (12.2) | 1.00 (reference) | 1.00 (reference) | 1.00 (reference) |
|  | Weaker (0-4) | 6,308 | 2,605 (41.3) | 3.36 (2.81, 4.02) | 2.57 (2.21, 2.99) | 2.59 (2.26, 2.98) |

Note: ^a^332 (3.7%) respondents missing; ^b^1,097 (12.2%) respondents missing.

**References**

1. Lundberg, O. & Nystrom Peck, M.N. A simplified way of measuring sense of coherence: experiences from a population survey in Sweden. *Eur. J. Public Health* **5**, 56-59. https://doi.org/10.1093/eurpub/5.1.56 (1995).

2. Chiesi, F., Bonacchi, A., Primi, C., Toccafondi, A. & Miccinesi, G. Are three items sufficient to measure sense of coherence? Evidence from nonclinical and clinical samples. *Eur. J. Psychol. Assess*. **34**, 229-237. https://doi.org/10.1027/1015-5759/a000337 (2018).
